# The Nitrogen Removal Characteristics of a Novel Salt-Tolerant Bacterium, *Enterobacter quasihormaechei* DGFC5, Isolated from Municipal Sludge

**DOI:** 10.3390/microorganisms12122652

**Published:** 2024-12-20

**Authors:** Bingguo Wang, Huanlong Peng, Wei Liu

**Affiliations:** 1 School of Environmental Science and Engineering, Sun Yat-Sen University, Guangzhou 510006, China; 2 Guangdong Provincial Key Laboratory of Environmental Pollution Control and Remediation Technology, Guangzhou 510006, China; 3 Institute of Agricultural Resources and Environment, Guangdong Academy of Agricultural Sciences, Guangzhou 510640, China

**Keywords:** heterotrophic nitrification–aerobic denitrification, salt-tolerant, nitrogen metabolism pathway, environmental factors

## Abstract

A novel bacterial strain, *Enterobacter quasihormaechei* DGFC5, was isolated from a municipal sewage disposal system. It efficiently removed ammonium, nitrate, and nitrite under conditions of 5% salinity, without intermediate accumulation. Provided with a mixed nitrogen source, DGFC5 showed a higher utilization priority for NH_4_^+^-N. Whole-genome sequencing and nitrogen balance experiments revealed that DGFC5 can simultaneously consume NH_4_^+^-N in the liquid phase through assimilation and heterotrophic nitrification, and effectively remove nitrate via aerobic denitrification and dissimilatory reduction reactions. Single-factor experiments were conducted to determine the optimal nitrogen removal conditions, which were as follows: a carbon-to-nitrogen ratio of 15, a shaking speed of 200 rpm, a pH of 7, C_4_H_4_Na_2_O_4_ as the carbon source, and a temperature of 30 °C. DGFC5 showed efficient nitrogen purification capabilities under a wide range of environmental conditions, indicating its potential for disposing of nitrogenous wastewater with high salinity.

## 1. Introduction

With the increasing level of global industrialization in recent years, large quantities of nitrogenous substances have been developed and used as raw materials in production, leading to a rapid rise in the nitrogen concentration of discharged wastewater. The excessive emission of nitrogen compounds causes various environmental issues, such as water quality deterioration and eutrophication, posing significant threats to normal human activities [1,2,3]. Biological nitrogen removal has been adopted globally due to its technical and economic advantages compared to conventional physical and chemical treatment methods [4]. However, traditional biological denitrification requires the simultaneous use of nitrifying and denitrifying bacteria, which have distinct differences in their requirements for dissolved oxygen, organic matter, and environmental factors, necessitating the conduction of nitrification and denitrification in two separate reactors under different conditions [5,6]. Moreover, as autotrophic microorganisms, nitrifying bacteria have disadvantages such as slow growth and a low tolerance to extreme conditions, particularly in wastewater with a high chemical oxygen demand (COD), in which the rapid growth of heterotrophic bacteria inhibits the proliferation of nitrifying microorganisms [7,8]. The recirculation of nitrified liquid and sludge required in this process also results in higher economic costs and technical requirements. With the emergence of simultaneous nitrification and denitrification (SND) methods, it is now possible to use a single bacterium to perform both nitrification and denitrification, thereby achieving nitrogen removal in wastewater within one reactor [9,10]. Extensive research has led to the discovery and isolation of plenty of heterotrophic nitrifying–aerobic denitrifying (HNAD) microorganisms for use in nitrogen pollutant purification in wastewater. At present, the reported HNAD strains include *Candida boidinii* L21 [11], *Acinetobacter* sp. ND7 [12], and *Aeromonas* sp. HN-02 [13]. Not only do these strains have higher treatment efficiencies and greater adaptability than traditional anaerobic denitrifying microorganisms, but they also possess distinct denitrification properties. For example, *Pseudomonas* sp. XF-4 can effectively remove Cd(II) from wastewater with Cd concentrations of <10 mg/L without significantly affecting its nitrification and denitrification performances [14]. Under the condition of a C/N ratio of 10, *Pseudomonas* sp. LW60 can completely remove ammonia from wastewater at 4 °C [3].

It is noteworthy that most currently available HNAD bacteria tend to thrive in low-salinity environments, and their denitrification efficiency and growth rate significantly decrease when the salinity exceeds 3% [11,15]. The primary reason for this is that high salinity leads to high osmotic pressure, causing bacterial dehydration and reduced activity in metabolism-related enzymes, thereby inhibiting normal physiological functions and possibly leading to cell expiration [16], making such bacteria ineffective in the treatment of domestic sewage in coastal areas and high-salinity industrial wastewater such as that resulting from printing, dyeing, papermaking, aquatic processing, and petroleum refining [17,18]. The current processing techniques for such wastewater involve inserting a pre-desalination unit or adding fresh water to reduce the salinity through dilution, which increases the complexity of the system, as well as investment and operation costs. Therefore, screening and isolating an HNAD strain with both salt tolerance and efficient nitrogen removal capability is an effective strategy for addressing this challenge.

In this study, a salt-tolerant bacterial strain, *Enterobacter quasihormaechei* DGFC5, was successfully isolated from a municipal sewage disposal system. It achieved a superior nitrogen removal efficiency under 5% salinity. Further analysis using whole-genome sequencing and nitrogen balance revealed the nitrogen metabolism pathway of DGFC5. In addition, the influences of the carbon source, C/N ratio, dissolved oxygen, temperature, and pH on the heterotrophic nitrification by DGFC5 were evaluated. The results demonstrate that this strain not only maintains an excellent tolerance and HNAD capability under high salinity, but also has a broad adaptability to environmental conditions, indicating its potential in practical applications of high-salinity wastewater nitrogen removal.

## 2. Materials and Methods

### 2.1. Sediment Samples and Medium Preparation

The bacterial source was obtained from activated sludge from a municipal sewage disposal system which mainly uses the A2/O process to treat domestic sewage in Dongguan City (Guangdong, China). The moisture content and pH of the municipal sludge were 88.27% and 6.98, respectively. The sludge was transported to the laboratory using polypropylene containers and stored in a refrigerator at 4 °C. The following media were prepared to isolate and enrich the salt-tolerant HNAD bacteria. The enrichment medium was lysogeny broth (LB), consisting of 10 g/L peptone, 5 g/L sodium chloride, 1 g/L glucose, and 5 g/L yeast extract, and it had a pH range of 6.8–7.2. The nitrification medium (NM) contained 10.13 g/L C_4_H_4_Na_2_O_4_, 1.5 g/L KH_2_PO_4_, 4 g/L Na_2_HPO_4_, 0.098 g/L MgSO_4_, 0.944 g/L (NH_4_)_2_SO_4_, 2 mL/L trace element solution, and 50 g/L NaCl (salinity of 5%). Denitrification medium 1 (DM1) contained 10.13 g/L C_4_H_4_Na_2_O_4_, 1.5 g/L KH_2_PO_4_, 4 g/L Na_2_HPO_4_, 0.098 g/L MgSO_4_, 0.986 g/L NaNO_2_, 2 mL/L trace element solution, and 50 g/L NaCl (salinity of 5%). Denitrification medium 2 (DM2) contained 10.13 g/L C_4_H_4_Na_2_O_4_, 1.5 g/L KH_2_PO_4_, 4 g/L Na_2_HPO_4_, 0.098 g/L MgSO_4_, 1.214 g/L NaNO_3_, 2 mL/L trace element solution, and 50 g/L NaCl (salinity of 5%). DM1 was used to assess the strain’s capacity for nitrite removal, while DM2 was used to assess its nitrate removal ability. The trace element solution comprised 70.53 g/L EDTA-2Na·2H_2_O, 3.92 g/L ZnSO_4_·7H_2_O, 5.06 g/L MnCl_2_·4H_2_O, 1.61 g/L CoCl_2_·6H_2_O, 5.0 g/L FeSO_4_·7H_2_O, 5.5 g/L CaCl_2_, 1.57 g/L CuSO_4_·5H_2_O, and 1.1 g/L Na_2_MoO_4_·2H_2_O. Regarding the preparation of the screening medium, 0.1 mL of bromothymol blue (BTB) and 2 g of agar were added to the NM. All of the media were adjusted to a pH of 7 using 0.2 mol/L NaOH or 0.2 mol/L HCl and were autoclaved for 30 min at 121 °C before being used.

### 2.2. Microbial Enrichment, Isolation, and Screening

Enrichment and screening were performed to obtain strains with salinity tolerance and HNAD potential from the activated sludge. A sufficient amount of sludge was inoculated in enrichment medium for 24 h. Then, 3 mL of the bacterial suspension was transferred into 100 mL of NM and incubated at 30 °C and 150 rpm until turbidity occurred. We reinoculated 3 mL of the bacterial suspension into 100 mL fresh NM. This process was conducted in triplicate. The bacterial suspension was then diluted and spread on screening medium plates to isolate colonies with different morphologies and colors. The colonies were inoculated into the NM and DM2 and incubated for 48 h prior to analysis of the NO_3_^−^-N and NH_4_^+^-N concentrations. The strain with the best denitrification efficiency was selected for further study.

### 2.3. Strain Identification

The target strain was designated DGFC5. Shiyanjia Lab (Hangzhou, China) Co., Ltd. was entrusted to use the bacterial universal primers 27F (5′-AGAGTTTGATCCTGGCTCAG-3′) and 1492R (5′-GGTTACCTTGTTACGACTT-3′) for PCR amplification of the 16S rRNA gene of the strain, and the resulting product was sequenced using Sanger sequencing. After comparing by BLAST, a phylogenetic tree was constructed using the neighbor-joining method in MEGA software (Version 11.0.13). The nucleotide sequence data for the strain were deposited in the NCBI GenBank database (accession number: PQ366016).

### 2.4. Nitrogen Removal Capacity and Nitrogen Balance

Each of three 250 mL flasks containing 100 mL NM, DM1, or DM2 was inoculated with 2 mL of bacterial suspension (2% *v*/*v*) and incubated at 30 °C and 150 rpm for 48 h to evaluate the strain’s removal capability for the different nitrogen sources. The denitrification process and the effect of DGFC5 in mixed nitrogen sources ((NH_4_)_2_SO_4_, NaNO_3_, and NaNO_2_) under the same conditions were also studied. The ammonium, nitrate, and nitrite concentrations were maintained at the same values, with a total nitrogen concentration of 200 mg/L. Samples were collected from each medium every 6 h to measure the optical depth at 600 nm (OD600) and the total nitrogen (TN), NH_4_^+^-N, NO_3_^−^-N, and NO_2_^−^-N concentrations. All of the experiments were conducted in triplicate. We subtracted the NH_4_^+^-N, NO_2_^−^-N, and NO_3_^−^-N from the TN to calculate the organic nitrogen content. The intracellular nitrogen content was determined by elemental analysis. The specific method involves taking an appropriate amount of 48 h medium and collecting the bacteria after centrifugation. The bacteria were dried by a vacuum freeze dryer (FD-1-50+, BIOCOOL, Beijing, China), and then an elemental analyzer (Elementar Vario EL, ELTRA, Berlin, Germany) was used to detect the N content. The gaseous nitrogen loss was calculated by subtracting the TN and the intracellular nitrogen from the initial nitrogen concentration. The formula is N_g_ = N_0_ − TN_t_ − N_i_, where N_g_ is the gaseous nitrogen, N_0_ is the initial nitrogen concentration, and TN_t_ and N_i_ represent the TN and the intracellular nitrogen content at 48 h, respectively.

### 2.5. Genomic Analysis

DGFC5 was cultured in an enrichment medium for 24 h, and an appropriate amount of bacterial suspension was collected via centrifugation by a centrifuge (KH20R, Kaida, Changsha, China) operating at 10,000 rpm for 10 min at 4 °C. The genomic sequencing was performed by Shanghai Majorbio Bio-Pharm Technology Co., Ltd. (Shanghai, China) using Illumina (San Diego, CA, USA) sequencing. The obtained sequences were subjected to quality control using the fastp v0.20.0 software and were assembled using the SOAPdenovo v2.04 software. The gene annotation and function prediction were conducted by using the Kyoto Encyclopedia of Genes and Genomes database (KEGG). The genome data for the strain were deposited in the NCBI GenBank database (BioProject: PRJNA1198030).

### 2.6. Effects of Environmental Factors on Denitrification Efficiency of DGFC5

We investigated the influences of five factors—carbon source (glucose, sucrose, sodium acetate, sodium succinate, fumaric acid, and sodium citrate), C/N ratio (5, 10, 15, 20, and 25), shaking speed (50, 100, 150, 200, and 250 rpm), temperature (24, 26, 30, 33, and 36 °C), and pH (5, 6, 7, 8, and 9)—on the nitrogen removal efficiency of DGFC5. Specifically, 2 mL of bacterial suspension was inoculated into 100 mL of NM (2% *v*/*v*) and incubated at 30 °C and 150 rpm for 48 h, and samples were collected to measure the OD600 and the NH_4_^+^-N concentration. During the whole process conditions were kept consistent, except for the independent variable being tested. Each experiment was conducted in triplicate.

### 2.7. Analytical Methods

A spectrophotometer (T6 New Century, METASH, Shanghai, China) was used to determine the OD600 of bacterial cultures. According to the standard methods, the TN, NH_4_^+^-N, NO_2_^−^-N, and NO_3_^−^-N concentrations were determined using the alkaline persulfate digestion ultraviolet (UV) spectrophotometric method at 220 nm and 275 nm, the salicylic acid spectrophotometric method at 697 nm, the N-(1-naphthyl)-ethylenediamine spectrophotometric method at 540 nm, and the UV spectrophotometric method at 220 nm and 275 nm, respectively [19]. The pH and dissolved oxygen were measured using a digital pH meter (PH-100B, LiChen, Shanghai, China) and a dissolved oxygen meter (SX725, Sanxin Precision Instruments, Shanghai, China). The Gram-stained DGFC5 sample was observed using an optical microscope (LED510, DEZZ, Chongqing, China), and the microbial morphology was observed via scanning electron microscopy (SU8010, HITACHI, Tokyo, Japan).

## 3. Results and Discussion

### 3.1. Isolation and Identification of DGFC5

**Figure 1:**
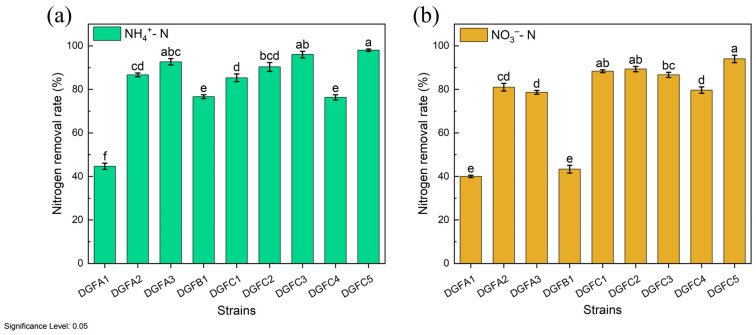
(**a**) The heterotrophic nitrification and (**b**) aerobic denitrification performances of the nine preliminarily selected strains within 48 h. The letters above the columns are used to show the significant difference (*p* < 0.05).

**Figure 2:**
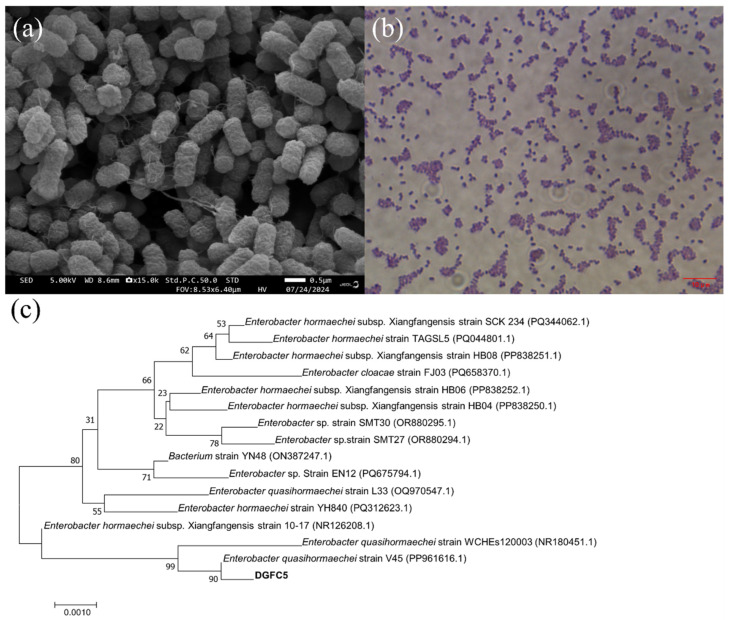
(**a**) A scanning electron micrograph of DGFC5, (**b**) the Gram staining results, and (**c**) the neighbor-joining phylogenetic tree of DGFC5 and its related bacteria. The number of bootstrap replications is 1000 and the bootstrap values are indicated at the branch nodes. The scale bar represents a 0.1% sequence divergence.

Following enrichment and isolation, nine salt-tolerant HNAD bacteria were screened from the sludge. Figure 1 shows that all of the strains were capable of removing ammonium and nitrate to varying extents. Further analysis revealed that under the same conditions, DGFC5 exhibited excellent nitrogen removal efficiency compared to the other strains, achieving final ammonium and nitrate removal rates of 98.43% and 94.65%, respectively; thus, it was selected for further study. The morphological observations revealed that DGFC5 formed circular colonies with a diameter of approximately 1 mm, an off-white color, a smooth surface, and regular edges, and it was identified as a Gram-positive bacterium. The 16S rRNA gene analysis confirmed that DGFC5 belongs to *Enterobacter quasihormaechei*, and the constructed phylogenetic tree and BLAST homology analysis showed that DGFC5 clustered with the *Enterobacter quasihormaechei* strain WCHEs120003, with a similarity of 99.72% (Figure 2).

### 3.2. Nitrogen Removal Characteristics of DGFC5

#### 3.2.1. Removal of NH_4_^+^-N by DGFC5

**Figure 3:**
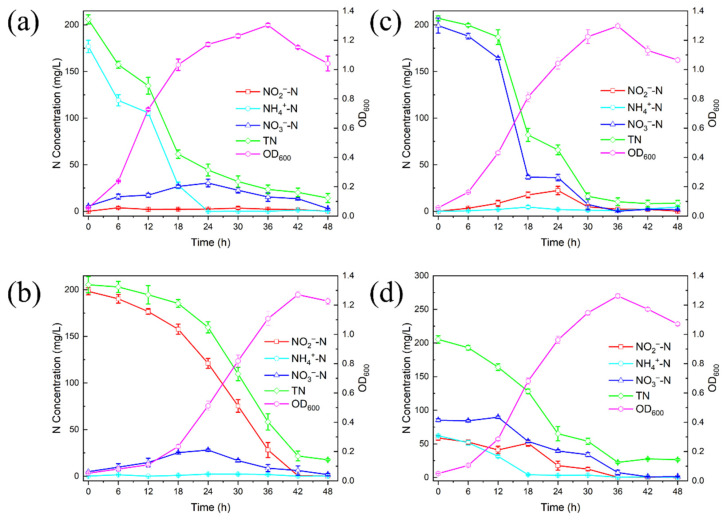
Nitrogen removal and growth characteristics of DGFC5 under 5% salinity conditions. (**a**) Nitrogen source corresponding to ammonium, (**b**) nitrogen source corresponding to nitrite, (**c**) nitrogen source corresponding to nitrate, and (**d**) ammonium, nitrite, and nitrate as mixed nitrogen sources.

The utilization of ammonia and the growth of DGFC5 when ammonium sulfate was the sole nitrogen source in the medium are depicted in Figure 3a. The OD600 of the strain gradually increased, reaching a maximum value of 1.303 at 36 h, followed by a decrease. The concentration of ammonia in the medium rapidly declined to 1.33 mg/L within 24 h, achieving nearly complete removal, after which it remained stable. The final nitrogen removal rate was 99.24%. The reduction trend of TN mirrored that of ammonia, i.e., an apparent decrease during the first 24 h followed by a gradual descent. Nitrite was almost undetectable throughout the process, whereas nitrate accumulated significantly, peaking at 30.33 mg/L at 24 h and then gradually decreasing. This phenomenon is the same as in the outcome of Ren et al. [20], suggesting that this strain gradually converts ammonia into nitrate via heterotrophic nitrification. However, in contrast to *Acinetobacter* sp. JR1 [15] and *Pseudomonas fluorescens* 2P24 [21], DGFC5 showed no nitrate accumulation during the entire denitrogenation process.

#### 3.2.2. Removal of NO_2_^−^-N by DGFC5

The utilization of nitrite and the growth of DGFC5 when only sodium nitrite was added to the medium are shown in Figure 3b. The OD600 of the strain reached a peak value of 1.270 at 42 h. During the reaction, the concentration of nitrite gradually decreased with time, reaching 0.5 mg/L at 42 h, and the final treatment rate was 99.74%. The nitrite purification rate was highest between 30 and 36 h, reaching 7.87 mg/(L·h), with an average purification rate of 4.13 mg/(L·h). The denitrification efficiency was significantly superior to those of *Acinetobacter indicus* ZJB20129 and *Bacillus thuringiensis* WXN-23 [22,23], indicating that DGFC5 possesses high nitrogen removal potential and a strong tolerance to nitrite. In addition, nitrate gradually accumulated after the reaction began, reaching a peak of 28.26 mg/L at 24 h and then declining to 1.70 mg/L, whereas the amount of ammonia did not significantly change.

#### 3.2.3. Removal of NO_3_^−^-N by DGFC5

The utilization of nitrate and the growth of DGFC5 when only sodium nitrate was added to the medium are shown in Figure 3c. This strain exhibited rapid proliferation over time, and the OD600 achieved a peak value of 1.296 at 36 h. The nitrate concentration declined slowly from 0 to 12 h after inoculation, and then it significantly decreased from 164.17 mg/L to 36.73 mg/L at 18 h, corresponding to a purification rate of 21.24 mg/(L·h). The nitrate concentration then stabilized for the next 6 h and continued to decrease after 24 h, reaching 1.83 mg/L at 48h, with a removal rate of 99.08%. It is worth noting that nitrite accumulation occurred during the reaction, but it gradually decreased until it was no longer detected after 24 h. This is different from several HNAD bacteria that have been reported, which proves that the strain has the capabilities of tolerance to and transformation of nitrite and it does not produce secondary pollution [9]. The curve of the total nitrogen was similar to that of nitrate. No significant changes in the ammonia concentration were observed, but a slight augmentation was noted after 36 h, potentially due to cell death and the release of intracellular nitrogen after the carbon sources were depleted [13,24].

Based on the experimental results, DGFC5 achieved ammonia, nitrite, and nitrate removal rates of >95%, demonstrating an excellent heterotrophic nitrification–aerobic denitrification performance. These results are the same as those of Ke et al. [22], but differ from those for *Paracoccus denitrificans* XW11 and *Sphingopyxis* sp. CY-10 [17,25], which have lower nitrite removal efficiencies than ammonia and nitrate nitrogen removal efficiencies, which may be due to the toxic effects of high nitrite concentrations on microorganisms. This observation is supported by Barathi et al. [26], who found that bacterial populations decreased significantly at nitrite concentrations of 60 mg/L as the concentrations of extracellular polymeric substances (EPSs) increased, suggesting that cell aggregation depends on protective responses.

#### 3.2.4. Removal of Mixed Nitrogen Sources by DGFC5

The growth and the nitrogen metabolism of DGFC5 when the medium contained a mixture of three inorganic nitrogen sources—namely, ammonia, nitrite, and nitrate—are presented in Figure 3d. The growth pattern was similar to that observed under single nitrogen source conditions, with the OD600 gradually increasing over time, reaching a maximum value of 1.262 at 36 h, and then decreasing. During the reaction, ammonia was removed first, with a purification rate of 93.32% at 18 h and an average purification rate of 3.22 mg/(L·h), which was less than the rate in the NM medium (8.27 mg/(L·h)). The trend of the nitrate concentration differed from that in the DM2 medium, i.e., the NO_3_^−^-N increased from the initial 85.21 mg/L to 89.71 mg/L at 12 h, which may have been due to the accumulation of nitrate during the heterotrophic nitrification of the ammonia. Subsequently, the nitrate concentration decreased slowly, achieving a removal rate of 98.72% at 42 h, at which time it was almost entirely removed, with an average purification rate of 2.00 mg/(L·h). The curve of the nitrite concentration was similar to that of the nitrate, initially increasing and then decreasing, reaching 22.33 mg/L at 18 h, then rapidly decreasing. It decreased to below the detection limit at 36 h and was then completely consumed. Throughout the reaction, the trend of the TN reduction was consistent with the trends of the reductions in ammonia, nitrite, and nitrate, resulting in a final removal rate of 87.12%. The final ammonia, nitrite, and nitrate removal rates were 99.80%, 99.81%, and 98.54%, respectively. These data suggest that DGFC5 exhibits a clear preference for utilizing ammonia over nitrite and nitrate, which may be due to its higher enzyme activity levels during heterotrophic nitrification than aerobic denitrification, leading to faster absorption and conversion of ammonia [27].

### 3.3. Analysis of Nitrogen Balance for DGFC5

The changes in the nitrogen concentration during the denitrification process of DGFC5 are presented in Table 1. Nitrogen balance calculations indicate that when NH_4_^+^-N was the sole nitrogen source, this strain primarily removed ammonium through an assimilatory proliferation process. After 48 h, 58.82% of the initial nitrogen source had been converted into intracellular nitrogen, supporting bacterial growth and anabolic processes, while only 34.22% of the ammonium was converted into gaseous nitrogen via the denitrification pathway. This phenomenon is similar to that observed for *Paracoccus versutus* LYM [28], which converted 49.7% of the ammonium into biomass; for *Acinetobacter calcoaceticus* HNR [29], which assimilated 52.1% of the ammonium into biomass; and for *Vibrio* sp. Y1-5 [30], which removed 83.18% of the ammonium via assimilation. When nitrite was the only nitrogen source, simple calculations revealed that 25.72% of the initial nitrogen source was converted into biomass, while 65.70% was removed from the liquid phase as gaseous nitrogen through aerobic denitrification. A similar pattern was observed when only nitrate was present. In other words, the N balance demonstrated that 63.13% of the nitrate was converted into gaseous nitrogen and only 29.78% was assimilated by the strain. These findings are consistent with those for other known strains, such as *Pseudomonas stutzeri* strain XL-2 [10], which converted 15.1% of nitrate into biomass, while 74.8% was transformed into gaseous nitrogen through multiple denitrification pathways. Additionally, *Pelomonas puraquae* WJ1 [31] eliminated approximately 74.95% of the initial nitrate as gaseous products. However, other studies have reported different results. For example, *Vibrio* sp. Y1-5 [30] primarily utilized nitrate assimilation, retained nitrogen within the cells, and removed approximately 98.0% of the nitrate within 48 h. *Pseudomonas stutzeri* T13 [32] was capable of completely transforming all of the nitrate when it was the sole nitrogen source, and 44.89% was assimilated as intracellular nitrogen, while 43.62% of the nitrate was reduced to nitrite, leading to accumulation and hindering its removal. These results suggest that DGFC5 is capable of HNAD under aerobic conditions, demonstrating its efficient removal of NH_4_^+^-N, NO_2_^−^-N, and NO_3_^−^-N. The genes and enzymatic systems related to HNAD played a crucial role throughout the procedure [33,34].

### 3.4. Analysis of Nitrogen Metabolic Pathway for DGFC5

**Figure 4:**
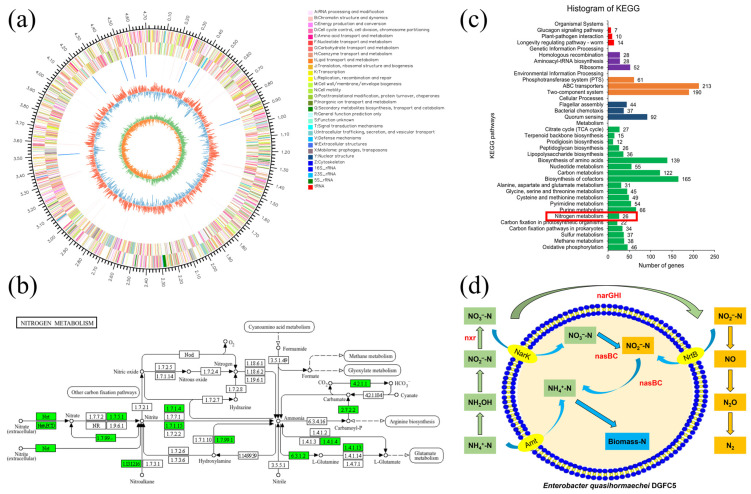
(**a**) A genetic map of DGFC5, (**b**) a nitrogen metabolism pathway map, (**c**) the KEGG pathway analysis, and (**d**) a schematic diagram of the nitrogen removal process. The green boxes indicate the genes present in the nitrogen metabolism pathway, and the numbers inside are the EC numbers of the key enzymes.

The genome size of DGFC5 is 4,742,570 bp. A total of 4381 genes were predicted, including 110 noncoding RNAs (25 rRNAs and 85 tRNAs), three CRISPR-Cas loci, and 17 genomic islands (Figure 4a). The functional genes related to the nitrogen metabolism of this strain are listed in Table S1. Through further comparison with the KEGG database, a nitrogen metabolic pathway map of the DGFC5 was constructed (Figure 4b). Extensive studies have been conducted on the HNAD pathway. During the HNAD process, ammonia is removed through both nitrification and denitrification stages. In the nitrification stage, ammonia is oxidized to hydroxylamine through the operation of ammonia monooxygenase (AMO), and then hydroxylamine is transformed to nitrite by hydroxylamine oxidase (HAO). Finally, it is converted into nitrate by nitrite oxidoreductase (NXR) [9,11]. During the denitrification stage, nitrate can be reduced to nitrite by nitrate reductase (NAP), which is subsequently transformed into NO through the operation of nitrite reductase (NIR). The NO is then further reduced to N_2_O and N_2_ by nitric oxide reductase (NOR) and nitrous oxide reductase (NOS), respectively [35]. Whole-genome analysis revealed that DGFC5 lacks the key genes *amoA* and *hao*, but possesses the *nxr* gene, suggesting that this strain may contain an as-yet-uncharacterized enzyme that is capable of directly oxidizing ammonia to nitrite. Previous research has demonstrated that *Pseudomonas citronellolis* YN-21 can efficiently remove ammonia under acidic conditions, even though it lacks the conventional nitrification genes *amoA* and *hao*. This is likely due to the occurrence of a nontraditional heterotrophic nitrification pathway [4]. Similar phenomena were described by Huang et al. [36] and Zhou et al. [37]. Moreover, genes such as *amt, glnA*, *glnK*, *gltBD*, and *gdhA* were also detected in DGFC5. These genes encode proteins that are crucial for regulating the uptake and assimilation of exogenous ammonia. This finding demonstrates the hypothesis that assimilation is one of the main pathways for ammonia removal by this strain, which is consistent with the observed increase in intracellular nitrogen content during ammonia removal [37,38].

In this study, DGFC5 was found to contain the genes *narGHI*, *nasBC*, *nirB*, and *nirD*. The *narGHI* genes encode nitrate reductase (NAR), which catalyzes the process of transforming nitrate to nitrite, which is the initial step of denitrification [4]. The *nasBC* genes encode assimilatory nitrate reductase (NAS), which can reduce nitrate to nitrite for use in anabolic processes [39,40]. Additionally, *nirB* and *nirD* encode nitrite reductase, which reduces nitrite to ammonia [41]. The current experiments have revealed that through the combined activity of these enzymes, DGFC5 can convert nitrate into ammonia via dissimilatory nitrate reduction (DNRA) and can subsequently remove ammonia via assimilation [42,43]. This suggests the existence of a nitrate removal pathway that is distinct from the conventional aerobic denitrification process for DGFC5. Furthermore, genes such as *nrtABC* and *nark*, which are involved in the transport of exogenous nitrogen, were also identified in this strain. The genes *napA*, *nir*, and *nos* were not detected, and only *norR* and *norV* were detected. However, gaseous nitrogen production was observed in the nitrogen balance experiments in which NO_2_^−^-N and NO_3_^−^-N were the sole nitrogen sources, suggesting that DGFC5 may possess an unknown nitrogen metabolism mechanism, which warrants further investigation.

### 3.5. Influences of Environmental Factors on Heterotrophic Nitrification by DGFC5

High concentrations of ammonia are a major pollutant in wastewater, posing significant challenges to wastewater treatment processes [12]. Therefore, NH_4_^+^-N was selected as the treatment target for DGFC5, and various environmental factors were evaluated under controlled conditions to explore their impacts on its heterotrophic nitrification.

#### 3.5.1. Carbon Sources

**Figure 5:**
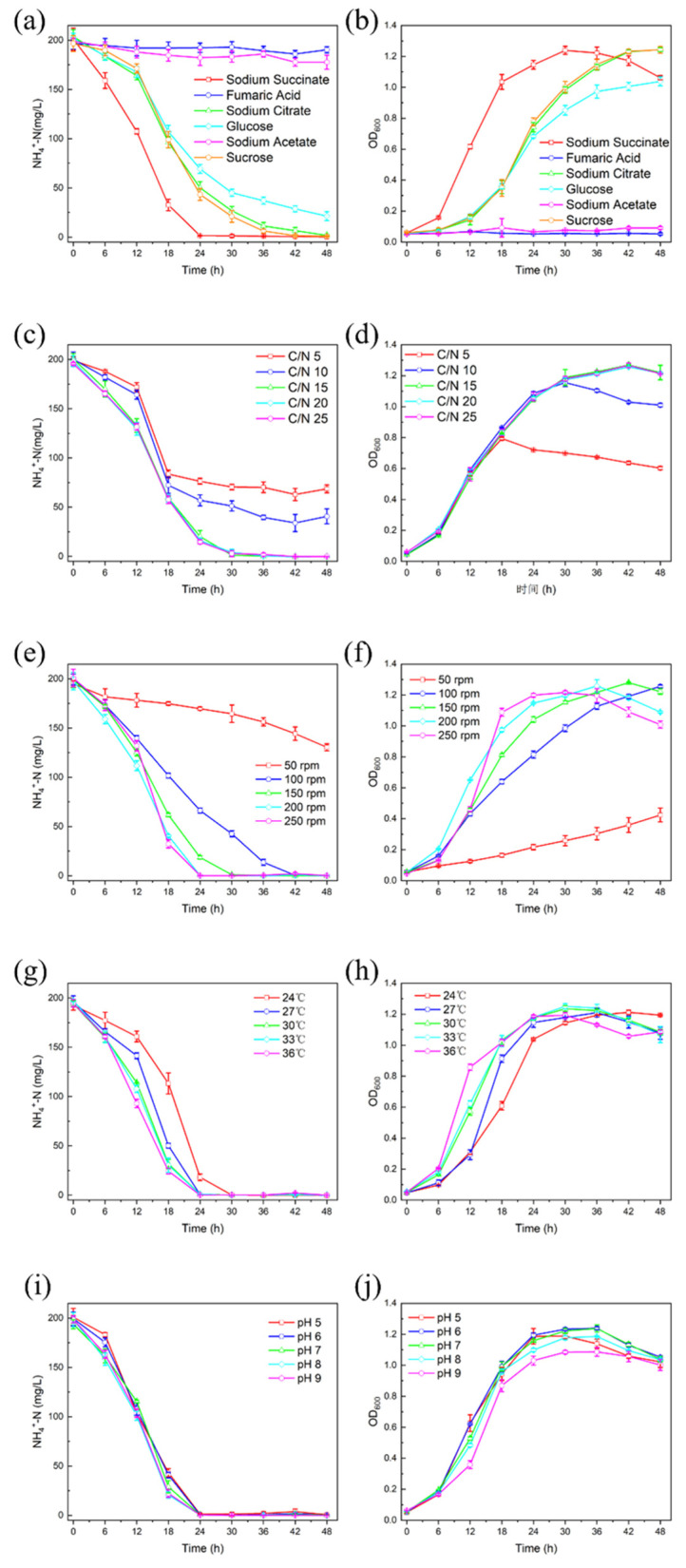
(**a**,**b**) The influences of different carbon sources, (**c**,**d**) C/N ratios, (**e**,**f**) rotation speeds, (**g**,**h**) temperatures, and (**i**,**j**) pH values on the heterotrophic nitrification performance of DGFC5 at 5% salinity. The values are the mean ± SD (error bars) of three replicates.

Carbon sources play an irreplaceable role in the bacterial life cycle, serving as essential raw materials for cell wall and cytoplasm synthesis. For heterotrophic bacteria, carbon sources are crucial, acting as energy sources and electron donors required for normal growth and metabolism, significantly influencing ammonia utilization and removal [44]. Due to differences in their structures and properties, different organic carbon sources impact microbial metabolic functions to varying degrees, thereby altering the ammonia removal efficiency. In this study, we investigated the influences of different sole carbon sources—namely, sodium succinate, fumaric acid, sodium citrate, glucose, sodium acetate, and sucrose—on the heterotrophic nitrification performance of the studied strain. The results are shown in Figure 5a,b. When only sodium acetate or fumaric acid was added to medium, DGFC5 failed to grow, indicating its inability to utilize these compounds. Conversely, when sodium succinate, sodium citrate, glucose, or sucrose was applied as the sole carbon source, the NH_4_^+^-N purification efficiency exceeded 90% within 48 h, demonstrating that DGFC5 can efficiently utilize these four carbon sources to remove ammonia. Notably, when only sodium succinate was added, the NH_4_^+^-N purification reached 99.34% at 24 h, significantly surpassing that of the other groups. Additionally, the cell growth rate was more advantageous, which is similar to the outcomes of Ke et al. [22] and Liao et al. [6]. A plausible explanation for this is that organic acid salts have lower molecular weights and simpler structures than sugars, allowing them to be more easily utilized to generate energy in the tricarboxylic acid cycle (TCA) [45]. Hence, in subsequent investigations we chose sodium succinate as the optimal carbon source.

#### 3.5.2. C/N Ratio

In addition to the carbon source, a nitrogen source is essential to the synthesis of proteins, amino acids, nucleotides, and other key cellular components for the normal proliferation and metabolism of bacteria. The C/N ratio directly affects the denitrification efficiency of the strain. A low C/N ratio causes insufficient carbon sources, which significantly decreases the efficiency of heterotrophic nitrification. In contrast, a high C/N ratio leads to incomplete utilization of the carbon source, increasing the COD level in the system. As shown in Figure 5c,d, the NH_4_^+^-N purification efficiencies for C/N ratios of 5 and 10 were 65.49% and 79.70%, respectively, and the OD600 value was <1.20, indicating that the insufficient carbon supply hindered further bacterial proliferation and nitrogen removal [46]. When C/N ratios increased to 15, 20, and 25, the NH_4_^+^-N removal efficiencies at 48 h were 99.34%, 99.78%, and 99.04%, respectively, achieving complete NH_4_^+^-N removal and similar cell growth trends. Furthermore, Sun et al. [47] reported that excessive carbon concentrations can adversely affect normal microbial activities. The results indicate that the optimal C/N ratio for denitrification by DGFC5 is 15.

#### 3.5.3. Dissolved Oxygen

Dissolved oxygen (DO) is important to the proliferation of aerobic bacteria, as it is an electron acceptor in the stepwise oxidation of NH_4_^+^-N to NO_3_^−^-N during heterotrophic nitrification. The DO content of the medium significantly affects the proliferation and N removal process of the bacteria. In our experiments, the DO concentration was varied by controlling the rotation speed to assess its impact on the NH_4_^+^-N purification efficiency of DGFC5. The DO concentrations of the media were 2.79, 3.15, 6.88, 7.28, and 7.50 mg/L at rotation speeds of 50, 100, 150, 200, and 250 rpm, respectively. Under a rotation speed of 50 rpm, the NH_4_^+^-N purification efficiency and the OD600 at 48 h were only 33.09% and 0.424, respectively, which was likely due to the insufficient DO concentration caused by the low rotation speed (Figure 5e,f). Increasing the rotation speed to 100 rpm resulted in complete substrate consumption within 42 h, while at 150 rpm, 99.53% of the NH_4_^+^-N was purified within 30 h. At rotation speeds of 200 and 250 rpm, there was no obvious difference in the nitrogen purification efficiency of DGFC5, and complete NH_4_^+^-N removal was achieved within 24 h. This may be because the increase in DO concentration with the increasing rotation speed significantly enhanced the activity levels of enzymes of nitrogen metabolism, thereby increasing the conversion efficiency of NH_4_^+^-N. Additionally, higher rotation speeds facilitated sufficient contact between the microorganisms and substrates, enhancing the efficient absorption and utilization of NH_4_^+^-N in the liquid phase. However, numerous studies have shown that excessively high rotation speeds weaken nitrogen metabolism functions, possibly due to inhibition of the nitrogen removal capability caused by excessive DO levels or the adverse effects of vigorous agitation on normal bacterial growth [15,20]. Thus, maintaining an appropriate DO level is essential for HNAD microorganisms to sustain normal physiological activity levels [48,49]. The outcomes also demonstrate that DGFC5 exhibits excellent oxygen tolerance and a good potential for NH_4_^+^-N removal under high DO conditions. Based on these findings, a rotation speed of 200 rpm was chosen for use.

#### 3.5.4. Temperature

An optimal temperature is critical for ensuring that bacteria can conduct normal physiological processes, primarily due to its influence on enzyme activity. Extreme temperatures can be detrimental to bacterial growth and can reduce the nitrogen removal performance. To investigate the temperature tolerance and optimal conditions of DGFC5, the strain was cultured at 24, 27, 30, 33, and 36 °C, and the NH_4_^+^-N removal was evaluated (Figure 5g,h). Within the temperature range of 24–36 °C, DGFC5 completely removed the NH_4_^+^-N within 48 h. At 24 °C, a lag phase of 6 h was evident, which was possibly due to the reduced enzyme activity related to nitrification at lower temperatures. Although DGFC5 achieved rapid nitrogen removal at 36 °C, the maximum OD600 value was lower (1.19) than those at 30 °C and 33 °C, indicating that these high temperatures inhibited bacterial growth and reproduction. Thus, the best temperature for DGFC5 was determined to be 30 °C, potentially due to the enhanced enzyme activity and increased ammonium content in the liquid phase at this temperature [50]. Similar outcomes have been shown in previous studies [20,51,52]. In summary, DGFC5 exhibited strong temperature adaptability.

#### 3.5.5. Initial pH

The current experiments have demonstrated that the pH significantly affects bacterial activity and material utilization by altering three aspects. First, the pH can change the electrical properties of cell membranes, impacting transmembrane transport. Second, the pH can induce changes in the charges of macromolecules such as proteins, thus disrupting normal physiological processes. Third, the pH can alter the properties of exogenous substances, making them less accessible for bacterial absorption. Therefore, in this study, we investigated the N removal efficiency and growth of DGFC5 under pH conditions of 5–9. Figure 5i,j show that the heterotrophic nitrification performance of DGFC5 was similar across the pH range of 5–9, with rapid reductions in the NH_4_^+^-N content within 24 h. Moreover, this strain achieved final nitrogen removal efficiencies greater than 98%, demonstrating its high NH_4_^+^-N removal capability over a relatively wide pH range, thus exhibiting significant practical application potential. In terms of its growth, the OD600 values were lower at pH values of 5 and 9 (1.18 and 1.09), which was likely due to the inhibitory effects of the acidic and alkaline conditions, respectively, on bacterial growth and proliferation. The pH has a significant effect on pollutant removal efficiency in wastewater [53], and in general, most HNAD bacteria thrive in neutral environments [54]. Therefore, a pH of 7 was determined to be the most suitable pH condition for DGFC5. A comprehensive comparative analysis of DGFC5 and other HNAD bacteria is presented in Table 2. Obviously, the NH_4_^+^-N removal efficiency of the DGFC5 strain under high salinity conditions is similar to that of most conventional HNAD bacteria, which proves that DGFC5 has excellent salt tolerance and nitrogen removal ability.

## 4. Conclusions

The *Enterobacter quasihormaechei* DGFC5 strain, which has an excellent HNAD ability under high salinity conditions, was isolated and screened from municipal sludge. DGFC5 efficiently removed NH_4_^+^-N, NO_3_^−^-N, and NO_2_^−^-N from the wastewater without the accumulation of intermediate products. In addition, DGFC5 can tolerate high amounts of NO_2_^−^-N stress and maintain an excellent N removal efficiency and removal rate under high salinity stress. Besides, in addition to wastewater treatment, it may also be used in the ecological restoration of saline–alkali land. Whole-genome analysis revealed that this strain primarily removes NH_4_^+^-N via assimilation and converts nitric nitrogen into a gaseous product under aerobic conditions. The optimum nitrogen removal conditions of DGFC5 were as follows: a C/N ratio of 15, sodium succinate as the carbon source, a shaking speed of 200 rpm, a pH of 7, and a temperature of 30 °C. In addition, this strain achieved an outstanding N purification efficiency under a wide range of environmental conditions, indicating that DGFC5 has obvious advantages in treating wastewater with high salinity and a high nitrogen concentration.

## Figures and Tables

**Table 1:** Concentrations of different forms of nitrogen after 48 h of cultivation.

Substrate	Initial TN (mg/L)	Nitrogen Concentrations After 48 h (mg/L)				Intracellular Nitrogen (mg/L)	Gaseous Nitrogen (mg/L)
		NH_4_^+^-N	NO_2_^−^-N	NO_3_^−^-N	Organonitrogen		
Ammonia nitrogen	205.84 ± 4.94	0.16 ± 0.03	0.16 ± 0.02	3.05 ± 0.17	10.96 ± 4.85	121.07 ± 3.86	70.44 ± 6.43
Nitrite nitrogen	205.37 ± 8.62	0.13 ± 0.10	0.08 ± 0.01	1.70 ± 1.16	15.71 ± 1.54	52.82 ± 5.50	134.93 ± 7.66
Nitrate nitrogen	207.01 ± 5.65	4.67 ± 0.81	-	1.83 ± 0.67	8.17 ± 2.77	61.65 ± 3.13	130.69 ± 4.61

- denotes undetectability.

**Table 2:** NH_4_^+^-N removal capacity of different strains under optimal environmental conditions.

HNAD Strain Name	Optimum Carbon Source	Optimum Shaking Speed (rpm)	Optimum C/N	Optimum Temperature (°C)	Optimum pH	Salinity (%)	Initial NH4+-N (mg/L)	Reaction Time (h)	NH4+-N Removal Efficiency (%)	References
*Cupriavidus* sp. S1	Sodium pyruvate	-	14.0	-	-	0	100.00	36	99.68	[47]
*Pseudomonas citronellolis* YN-21	Succinate	-	10.0	30	5.0	0	100.00	18	100.00	[9]
*Halomonas venusta* SND-01	Sodium citrate	-	10.0	-	8.5	3	100.00	24	98.00	[36]
*Acinetobacter* sp. JR1	Fumaric acid	120 rpm	16.0	30	4.5	0	100.00	24	98.50	[15]
*Acinetobacter calcoaceticus* TY1	Sodium citrate	90 rpm	10.0	-	7.0	0	106.00	60	97.70	[34]
*Acinetobacter* sp. ND7	Sodium citrate	150 rpm	8.0	35	-	0	50.00	24	93.23	[12]
*Aeromonas* sp. HN-02	-	-	-	30	10.0	0	35.00	10	100.00	[13]
*Pseudomonas* sp. DM02	-	-	8.0	-	7.0	0	10.00	24	100.00	[51]
*Enterobacter quasihormaechei* DGFC5	Sodium succinate	200 rpm	15.0	30	7.0	5	198.33	48	100.00	In this study

## Data Availability

The original contributions presented in the study are included in the article/Supplementary Material, further inquiries can be directed to the corresponding authors.

## Supplementary Materials

The following supporting information can be downloaded at: https://www.mdpi.com/article/10.3390/microorganisms12122652/s1, Table S1. N metabolism-related functional genes in the genome of DGFC5.
