# Supplementary material for: The Nitrogen Removal Characteristics of a Novel Salt-Tolerant Bacterium, Enterobacter quasihormaechei DGFC5, Isolated from Municipal Sludge
Source: Microorganisms. 2024 Dec 20;12(12):2652. doi: 10.3390/microorganisms12122652 (PMC11728697; doi:10.3390/microorganisms12122652)
Supplement: Supplementary file 1 [file microorganisms-12-02652-s001.zip › microorganisms-3365181-supplementary.pdf]

Supplementary Materials

The Nitrogen Removal Characteristics of a Novel Salt-Tolerant Bacterium, *Enterobacter quasihormaechei* DGFC5, Isolated from Municipal Sludge

Bingguo Wang[1,2], Huanlong Peng[3], Wei Liu[1,2]*

[1]School of Environmental Science and Engineering, Sun Yat-Sen University, Guangzhou 510006, China

[2] Guangdong Provincial Key Laboratory of Environmental Pollution Control and Remediation Technology, Guangzhou 510006, China

[3]Institute of Agricultural Resources and Environment, Guangdong Academy of Agricultural Sciences, Guangzhou 510640, China

*Corresponding authors

E-mail: esslw@mail.sysu.edu.cn

Table S1. N metabolism-related functional genes in the genome of DGFC5

KO ID	Genes	Protein/Enzyme	Function
K15577	nrtABC	nitrate/nitrite transport system permease protein	Transport of NO_2^--N
K02575	narK	nitrate/nitrite transporter	Transport of NO_2^--N/NO_3^--N
K01915	glnA	glutamine synthetase	Synthesis of glutamine synthetase
K04752	glnK	nitrogen regulatory protein P-II	Synthesis of nitrogen regulatory proteins P-II
K00370	nxr	nitrate reductase/nitrite oxidoreductase	Oxidation of NO_2^--N to NO_3^--N
K00371	narGHI	nitrate reductase/nitrite oxidoreductase	Reduction of NO_3^--N to NO_2^--N
K00372	nasBC	assimilatory nitrate reductase	Reduction of NO_3^--N to NO_2^--N
K00363	nirBD	nitrite reductase	Reduction of NO_2^--N to NH_4^+-N
K00266	gltBD	glutamate synthase	Synthesis of glutamate synthase
K00262	gdhA	glutamate dehydrogenase	Synthesis of glutamate dehydrogenase
K03320	amt	ammonium transporter	Transport of NH_4^+-N
K12266	norR	anaerobic nitric oxide reductase transcription regulator	-
K12264	norV	anaerobic nitric oxide reductase flavorubredoxin	-
